# Periodontitis‐Associated Circulating EVs Promote Colorectal Cancer Progression via Carnosine‐Mediated Acidosis Adaptation

**DOI:** 10.1111/cpr.70254

**Published:** 2026-07-01

**Authors:** Ruoyi Wu, Zihan Cai, Hualing Sun, Haikun Yu, Bicheng Zhang, Chi Zhang, Yi Fan, Xiaoxuan Zhu, Yueqi Ni, Yu Cui, Kaixin Wang, Zhe Li, Xinyi Zhou, Qing He, Yanru Wu, Yufeng Zhang

**Affiliations:** ^1^ State Key Laboratory of Oral & Maxillofacial Reconstruction and Regeneration, Key Laboratory of Oral Biomedicine Ministry of Education, Hubei Key Laboratory of Stomatology, School & Hospital of Stomatology Wuhan University Wuhan China; ^2^ Medical Research Institute, School of Medicine Wuhan University Wuhan China

**Keywords:** colorectal neoplasms, extracellular vesicles, periodontal diseases, tumour microenvironment

## Abstract

Colorectal cancer (CRC) is the third most common malignancy worldwide. Epidemiological studies have suggested a positive association between periodontitis (PD) and CRC risk; however, the mechanistic basis underlying this relationship remains unclear. Extracellular vesicles (EVs) represent an important mode of systemic communication and may mediate the distal effects between PD and CRC. PD model was established in *Apc* ^*+/−*^ mice with spontaneous intestinal tumorigenesis. Tumour onset, burden, and progression were evaluated in the colorectum and small intestine. Circulating EVs were isolated from the plasma of PD or sham mice and characterised. The functional contribution of EVs was assessed using pharmacological inhibition of EV release and MC38 syngeneic tumour models. Metabolomic profiling, RNA sequencing, and in vitro functional assays were performed to investigate EV cargo and underlying mechanisms. PD significantly accelerated CRC onset and increased tumour number and size in *Apc* ^*+/−*^ mice. Inhibition of EV release by GW4869 attenuated PD‐driven tumour progression, indicating a critical role of periodontitis‐associated EVs (PDEVs). PDEVs promoted tumour growth and induced an immunosuppressive tumour microenvironment in MC38 transplanted tumours. Metabolomic analysis revealed marked enrichment of carnosine in PDEVs. Under acidic conditions, EV‐delivered carnosine alleviated intracellular acidosis, preserved lysosomal positioning and acidification, and promoted proliferation, migration, and epithelial–mesenchymal transition of MC38 cells. Collectively, circulating EV‐mediated metabolic communication pathway linking PD to CRC progression. By delivering carnosine, PDEVs support malignant phenotypes and facilitate tumour adaptation to acidic stress. Circulating EV‐associated carnosine may represent a potential biomarker and a candidate target for modulating CRC progression in high‐risk populations.

## Graphical abstract

This study identifies a link between periodontitis and CRC progression mediated by carnosine‐enriched circulating EVs. These EVs target the intestine and elevate tumour cell intracellular pH (pHi), which rescues lysosomal function. This process drives a transition from dysfunctional lysosomes to functional ones, promoting a high malignancy phenotype in colorectal cancer.

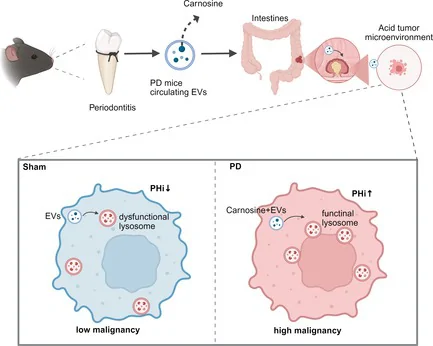

## 1 Introduction

Periodontitis (PD) is one of the most prevalent chronic inflammatory diseases of the oral cavity [1, 2]. With the growing recognition of the ‘oral–systemic’ health concept, accumulating evidence indicates that periodontitis‐associated persistent inflammation, microbial dysbiosis, and the translocation of periodontal pathogens and inflammatory mediators into the bloodstream can initiate or exacerbate a state of systemic low‐grade inflammation, thereby contributing to the onset and progression of multiple systemic disorders [3, 4, 5]. Both epidemiological and mechanistic studies further suggest that periodontitis is associated with increased risks of cardiovascular disease, diabetes mellitus, rheumatoid arthritis, and gastrointestinal malignancies [6, 7, 8, 9, 10, 11, 12].

PD and colorectal cancer (CRC) are highly prevalent diseases that significantly impact patients' psychological and physical health [13, 14, 15, 16, 17, 18]. Epidemiological studies have observed that individuals with periodontitis, particularly those with severe periodontal tissue destruction, may have an increased risk of developing colorectal cancer [9]. However, the precise biological mechanisms underlying this potential link remain poorly understood, creating a critical knowledge gap.

Extracellular vesicles (EVs) have emerged as critical mediators of systemic communication, which carry bioactive molecules (e.g., proteins, nucleic acids) to target cells, facilitating crosstalk between distant organs and driving pathological processes such as cancer [19, 20, 21, 22]. Previous studies have shown that circulating EVs derived from periodontitis patients can promote insulin resistance in rats [4], this evidence supports the notion that EVs indeed act as mediators linking periodontitis to other systemic diseases. In this context, whether PD associated circulating EVs contribute to the pro‐tumorigenic effects of PD on CRC, and what the underlying mechanisms might be, remain to be elucidated and warrant further investigation.

To investigate the role of circulating EVs from PD mice in the malignant progression of CRC, *Apc* ^+/−^mice and MC38 xenograft models were employed, confirming that PDEVs promote CRC progression. Further analysis revealed significant compositional differences between circulating EVs from PD mice (PDEVs) and healthy mice (HEVs), with Carnosine being highly enriched. However, in PD mice and patients, carnosine is packaged into circulating EVs, thereby escaping carnosinase degradation. This EV‐encapsulated carnosine can subsequently reach intestinal tissues and exert biological effects. Acting as a buffering metabolite [23], Carnosine facilitates the adaptation of MC38 cells to the acidic tumour microenvironment and mitigates lysosomal dysfunction. Lysosomal function is critical for solid tumours, as it mediates the degradation of MHC‐I molecules, thereby promoting tumour immune evasion [24]. Furthermore, data from the TCGA database indicate that high expression of lysosome‐associated genes correlates with poor prognosis in CRC patients. These findings advance our understanding of the EV‐mediated link between PD and CRC, offering potential therapeutic targets for intervention.

## 2 Materials and Methods

### 2.1 Animal Use and Care

C57BL/6J mice were purchased from GemPharmatech Co. Ltd. and *Apc* ^*+/−*^ mice were purchased from Cyagen Biosciences Inc. All mice were housed in specific pathogen‐free (SPF) facilities. At the end of the experiment, the mice were euthanized by cervical dislocation following intraperitoneal anaesthesia. Sample allocation, experimental procedures with outcome assessment, and data analysis were conducted independently by different members of the research team.

### 2.2 Establishment of Periodontitis Mice Model

For modelling, mice were anaesthetised by intraperitoneal injection of 1% sodium pentobarbital, followed by ligation of bilateral maxillary second molars with 5–0 silk sutures, while the ligatures were inspected and re‐ligated when necessary. The sham group mice underwent sham surgery following the above method, and the ligature wire was removed after the procedure.

### 2.3 Histological Analysis of Periodontitis in Mouse Mandibles

The mouse maxillae were fixed with neutral fixative (Servicebio, Wuhan, China), decalcified in 10% EDTA (pH 8.0) solution at room temperature for 1 month (with EDTA solution changed every 3 days), and then embedded in paraffin. Sagittal sections of 5 μm thickness along the tooth longitudinal axis were obtained. The sections (*n* = 3) were subsequently stained using an H&E staining kit (Servicebio, Wuhan, China) and a TRAP staining kit (Solarbio, China) according to the manufacturers' instructions.

### 2.4 Micro‐CT Scanning

The fixed mandibles (*n* = 3) were scanned by a Quantum GX microCT imaging system (PerkinElmer, USA). The CT was operated at 70 kV and 114 μA, with a scan time of 14 min and a pixel size of 20 μm.

### 2.5 Spontaneous Colorectal Cancer Model

Eight‐week‐old *Apc* ^*+/−*^ mice were randomly divided into periodontitis and sham groups, as well as periodontitis and periodontitis+GW4869 groups. The periodontitis model was established as described above. To inhibit the release of circulating EVs, GW4869 (i.p) was administered at a concentration of 2.5 mg/kg every 2 days until the end of the experiment as previously described [25]. The disease activity index (DAI) score was calculated weekly based on the mouse's faecal occult blood index, stool consistency, and weight loss. Intestinal and colorectal tissues were harvested when mice reached 24 weeks of age. The intestinal and colorectal tissues were photographed, and the number and size of tumours were measured. Tumour load was calculated as the sum of the diameters of all tumours in each mouse, as previously described [10].

### 2.6 Isolation and Characterisation of Circulating EVs in Mice

Circulating EVs were isolated from the plasma of C57BL/6 mice (Sham or PD, as previously described) using differential centrifugation. Briefly, whole blood was collected in EDTA‐coated tubes and subjected to sequential centrifugation at 1200 × g for 15 min (to remove cells) and 12,000 × g for 30 min (to remove platelets). The resulting supernatant was ultracentrifuged at 120,000 × g for 70 min to pellet EVs, which were then resuspended in PBS.

The isolated EVs were characterised as follows: nanoparticle tracking analysis (NTA) was performed to determine particle size distribution and concentration (ZetaView, ParticleMetrix); transmission electron microscopy (TEM) was used to assess EV morphology (JEM‐2100, JEOL); and Western blotting was conducted to verify EV marker proteins (*n* = 3) using the following primary antibodies: anti‐HSP90 monoclonal antibody (60318‐1‐Ig, Proteintech, China) and anti‐CD9 rabbit polyclonal antibody (A1703, ABclonal, China).

### 2.7 Tracking and Analysis of EVs Biodistribution

20 μg of mouse plasma EVs were diluted in 100 μL PBS and incubated with DIO dye (Beyotime, China) at a 1:200 dilution for 20 min at 37°C. The mixture was transferred to a 100 kDa ultrafiltration tube and centrifuged at 12,000 × g for 15 min. This washing procedure was repeated twice to remove excess dye.

To mimic the trafficking of circulating EVs in mice, the freshly labelled EVs (in a final volume of 100 μL PBS) were administered to mice via intravenous tail vein injection. Precisely 2 h post‐injection, the animals (*n* = 3) were euthanized, and intestinal tissues were promptly collected and fixed in 4% paraformaldehyde overnight. The fixed tissues were then embedded in OCT compound and cryo‐sectioned into 10 μm‐thick slices. Representative fluorescent images were captured using Leica Stellaris 5 WLL (Leica, Germany).

### 2.8 Metabolomic Profiling of EVs

After establishing the periodontitis model, mouse plasma was collected, and EVs were isolated as previously described. The metabolomic profiling of EVs was performed by Biomarker Technologies Corporation (*n* = 4). Briefly, the extraction solvent was added to the EVs, followed by centrifugation to collect the supernatant, which was then lyophilized. The dried samples were reconstituted and sonicated, then centrifuged to obtain the final supernatant for instrumental analysis.

The analysis was conducted using a Waters Acquity I‐Class PLUS ultra‐high‐performance liquid chromatography (UPLC) system coupled with a Waters Xevo G2‐XS QTOF high‐resolution mass spectrometer. Raw data acquired by MassLynx V4.2 were processed using Progenesis QI software for peak extraction, alignment, and other data processing steps. Metabolite identification was performed based on the METLIN database integrated within Progenesis QI, public databases, and the in‐house database of Biomarker Technologies.

### 2.9 Enzyme‐Linked Immunosorbent Assay (ELISA)

Plasma samples and EVs samples were diluted in PBS, and the EVs samples were lysed with RIPA buffer. A standard sandwich ELISA was performed using a Mice Carnosine ELISA Kit (Centralgene Biotechnology, China) according to the manufacturer's instructions. All samples were assayed in duplicate (*n* = 3), and concentrations were interpolated from the standard curve.

### 2.10 Release of EV‐Associated Carnosine by Freeze–Thaw Cycles

To promote the release of intravesicular cargo, including carnosine, purified EVs were subjected to repeated freeze–thaw cycles. EV suspensions were rapidly frozen in liquid nitrogen (−196°C) for 5 min and subsequently thawed at 37°C until completely liquefied. This freeze–thaw procedure was repeated three times.

### 2.11 Subcutaneous Tumour Models and Flow Cytometry Analysis

The MC38 cells were purchased from Servicebio Biotechnology Co. Ltd. and cultured in DMEM supplemented with 10% fetal bovine serum (FBS) and 1% penicillin–streptomycin at 37°C under 5% CO_2_. A total of 1 × 10^6^ MC38 cells were subcutaneously injected into the right flank of male C57BL/6J mice (*n* = 5). To evaluate the dose‐dependent effects of PDEVs and determine the optimal dosage for this study, mice were randomly assigned to different groups (*n* = 5 per group) and received peritumoral injections of Vehicle (PBS) or PDEVs at varying doses (10, 20 and 40 μg per injection). Starting on day 6 post‐inoculation, the treatments were administered via subcutaneous injection every 2 days. Based on the observed pro‐tumorigenic efficacy and previous studies [26], the 20 μg dose was selected for all subsequent experiments. For the comparative study, mice were treated with Vehicle, HEVs (20 μg), or PDEVs (20 μg) following the same administration schedule until the end of the experiment. Tumour size was measured every 2 days. After euthanasia, tumour tissues were excised and weighed. The experimental protocols were approved by the Institutional Animal Care and Use Committee of the Medical Research Institute of Wuhan University. The ethics approval number is MRI2024‐LAC182. According to the approved protocol, the maximum tumour burden permitted was 2000 mm^3^.

The MC38 transplanted tumours (*n* = 5) were dissociated into single cells, filtered through a 40 μm strainer, and counted. The samples were divided into aliquots of 5 × 10^6^ cells each. The cells were stained for viability and fluorescence using Zombie Violet Fixable Viability Kit (BioLegend, USA), Brilliant Violet 785 anti‐mouse CD45 Antibody (BioLegend, USA), APC anti‐mouse CD8a Antibody (BioLegend, USA), PE anti‐mouse NK‐1.1 (BioLegend, USA), and FITC anti‐mouse CD4 Antibody (BioLegend, USA). After incubation at room temperature for 30 min protected from light, the cells were centrifuged and the supernatant was discarded. The cells were then resuspended in PBS. Data were acquired using a Beckman CytoFLEX LX flow cytometer and analysed with FlowJo software.

### 2.12 RNA‐Seq

After euthanasia by cervical dislocation, subcutaneously transplanted tumours (*n* = 3) were harvested from the mice. The RNA‐seq service for the transplanted tumours was provided by Biomarker Technologies Corporation. Briefly, mRNA was enriched using oligo(dT)‐conjugated magnetic beads. The mRNA was then randomly fragmented by incubation with Fragmentation Buffer. Using the fragmented mRNA as a template, first‐strand cDNA was synthesized, followed by second‐strand cDNA synthesis, after which the double‐stranded cDNA was purified. The purified cDNA underwent end repair, A‐tailing, and adapter ligation. Size selection was performed using AMPure XP beads, and the cDNA library was enriched by PCR. After library construction, initial quantification was performed using a Qubit 3.0 Fluorometer, with a required concentration of ≥ 1 ng/μL. The insert size distribution was assessed using a Qsep400 High‐Throughput Analysis System. Once the insert size met expectations, the library's effective concentration (> 2 nM) was accurately quantified via qPCR to ensure quality. Following quality control validation, high‐throughput sequencing was performed in paired‐end (PE) 150 bp mode.

### 2.13 EVs Loading With Carnosine and LC–MS Detection

HEVs (50 μg) were incubated with carnosine (10 μg) at 37°C for 4 h and the resulting product was collected as CarEVs. The EVs were then diluted with PBS to remove unbound carnosine and centrifuged at 100,000 × g for 70 min. The supernatant was discarded, and the pelleted EVs were lysed with RIPA buffer by gentle pipetting for 1 min. Next, 100 μL of double‐distilled water and 300 μL of acetonitrile were added to the lysate, followed by protein precipitation at −20°C for 30 min. After centrifugation at 16,000 × g for 10 min at 4°C, the supernatant was collected and analysed using liquid chromatography–tandem mass spectrometry (ExionLC AD/QTRAP 6500+, AB Sciex, USA).

### 2.14 Cell Culture

The MC38 cell line was obtained from Servicebio Biotechnology Co. Ltd. (Wuhan, China) and maintained in DMEM basal medium (Gibco, USA) supplemented with 10% fetal bovine serum (CellBox, China), 1% non‐essential amino acids (NEAA; Procell, China), and 1% penicillin/streptomycin (P/S; Procell, China). Cells were cultured at 37°C under a humidified atmosphere of 95% air and 5% CO_2_. For specific experiments requiring acidic conditions, the pH of the culture medium was adjusted to 6.7 using HCl. According to the experimental groups, PBS, HEVs/PDEVs/CarEVs (1 μg/mL), free Carnosine (0.1 ng/mL, equivalent to the carnosine content in 1 μg/mL of PDEVs) or Bafilomycin A1 (20 nM) were added.

### 2.15 CCK‐8 Cell Proliferation Assay

Cells (1 × 10^4^/well) were seeded into 96‐well plates. After 24 h of incubation, cell proliferation was evaluated using the Cell Counting Kit‐8 (CCK‐8; C0038, Beyotime, China) following the manufacturer's instructions. Briefly, 10 μL of CCK‐8 solution was added to each well containing 100 μL of culture medium, and the plate was incubated at 37°C for 1 h, protected from light. Absorbance was measured at 450 nm using a microplate reader. Blank wells containing medium and CCK‐8, but without cells, were used for background subtraction. Data were presented as optical density (OD) values or normalised to the control group.

### 2.16 Colony Formation Assay

Cells were seeded in 6‐well plates at a density of 500 cells per well. The medium and supplements were refreshed every 48 h. The experiment was terminated on day 14. Cells were fixed with neutral fixative (Servicebio, Wuhan, China) for 30 min and stained with crystal violet (Servicebio, Wuhan, China). The stained colonies were photographed for documentation. Colony quantification was performed using ImageJ software (*n* = 3).

### 2.17 Cell Migration Assay (Transwell)

Cells (5 × 10^4^/well) were seeded into the upper chambers of Transwell inserts (3422, Corning, USA). The lower chambers were filled with medium adjusted to either pH 7.4 or 6.7, supplemented with PBS, HEVs or CarEVs (1 μg/mL). After 48 h of incubation, migrated cells were fixed with neutral fixative (Servicebio, Wuhan, China) for 30 min and stained with crystal violet (Servicebio, Wuhan, China). The stained cells were photographed and quantified using ImageJ software (*n* = 3).

### 2.18 Wound Healing Assay

Seed cells (1 × 10^6^/well) in a 6‐well plate to form a confluent monolayer. Create a scratch using a sterile pipette tip. Wash with PBS to remove debris and add serum‐free medium. Capture the initial image (*n* = 7). Incubate the plate and acquire images at 24 h. Quantify the migration using ImageJ software and calculate the percentage of wound closure.

### 2.19 EdU Cell Proliferation Assay

Cells (1 × 10^4^/well) were seeded in confocal dishes. After 24 h, cell proliferation was assessed using the BeyoClick EdU‐594 Cell Proliferation Kit (C0078S, Beyotime, China) following the manufacturer's protocol. Following EdU staining, cells were washed twice with HBSS and counterstained with DAPI (Beyotime, China) at room temperature for 5 min. After two additional HBSS washes, samples were stored protected from light. Images were acquired using a confocal laser scanning microscope and processed with LAS X software.

### 2.20 RNA Extraction and Quantitative Real‐Time PCR

Total RNA was extracted from MC38 cells (*n* = 3) using RNAiso Plus reagent (Takara, Japan). Reverse transcription was performed with the HiScript III RT SuperMix for qPCR (+gDNA wiper) kit (Vazyme, China). Quantitative real‐time PCR (qPCR) was conducted by QuantStudio 1 Plus (Thermo Fisher, USA) with SupRealQ Purple Universal SYBR qPCR Master Mix (U+) (Vazyme, China). Gene‐specific primers listed in Table 1 were used to analyse mRNA expression levels.

**TABLE 1:** qPCR primers.

Gene name	Primer type	Sequence (5′ to 3′)	Length (bp)
Mice‐N‐cad	Forward	AGGCTTCTGGTGAAATTGCAT	21
Mice‐N‐cad	Reverse	GTCCACCTTGAAATCTGCTGG	21
Mice‐E‐cad	Forward	CAGTTCCGAGGTCTACACCT	20
Mice‐E‐cad	Reverse	TGAATCGGGAGTCTTCCGAAAA	22
Mice‐Twist	Forward	GGACAAGCTGAGCAAGATTCA	21
Mice‐Twist	Reverse	CGGAGAAGGCGTAGCTGAG	19
Mice‐Snail	Forward	CACACGCTGCCTTGTGTCT	19
Mice‐Snail	Reverse	GGTCAGCAAAAGCACGGTT	19
Mice‐vimentin	Forward	CGTCCACACGCACCTACAG	19
Mice‐vimentin	Reverse	GGGGGATGAGGAATAGAGGCT	21
Mice‐Actin	Forward	GTGACGTTGACATCCGTAAAGA	22
Mice‐Actin	Reverse	GCCGGACTCATCGTACTCC	19
Mice‐TFEB	Forward	AAGGTTCGGGAGTATCTGTCTG	22
Mice‐TFEB	Reverse	GGGTTGGAGCTGATATGTAGCA	22
Mice‐CTSB	Forward	CAGGCTGGACGCAACTTCTAC	21
Mice‐CTSB	Reverse	TCACCGAACGCAACCCTTC	19
Mice‐Lamp1	Forward	CAGCACTCTTTGAGGTGAAAAAC	23
Mice‐Lamp1	Reverse	CCATTCGCAGTCTCGTAGGTG	21
Mice‐Lamp2	Forward	TGTATTTGGCTAATGGCTCAGC	22
Mice‐Lamp2	Reverse	TATGGGCACAAGGAAGTTGTC	21
Mice‐Rab27a	Forward	TCGGATGGAGATTACGATTACCT	23
Mice‐Rab27a	Reverse	TTTTCCCTGAAATCAATGCCCA	22
Mice‐Rab27b	Forward	TCGGGAAAAACGTGTGGTTTA	21
Mice‐Rab27b	Reverse	TTCAAGAAGCTCTGTTGACTGG	22

### 2.21 pH Measurement

Extracellular pH: Culture supernatant from MC38 cells was collected, and pH was measured with a pH meter (*n* = 7).

Intracellular pH: Cells were loaded with 10 μM BCECF AM (Beyotime, China) in 100 μL solution per well of a 96‐well plate and incubated at 37°C for 30–60 min. After removal of the dye solution, cells were washed twice with HHBS buffer. Fluorescence intensity was measured using a microplate reader at excitation/emission wavelengths of 490/535 nm and 440/535 nm (*n* = 7).

Calibration curve: Cells were incubated with calibration buffers in parallel at 37°C for 15 min, along with 10 μM nigericin (final concentration). Subsequent steps were performed as described above.

The intracellular pH of experimental samples was calculated by comparing their fluorescence ratios (490/440 nm) with the standard curve.

### 2.22 Lysotracker Red Staining and Imaging

MC38 cells pre‐seeded in confocal dishes were stained with Lysotracker Red dye (Beyotime, China) according to the manufacturer's instructions. Following staining, the dye solution was removed, and cells were washed twice with HBSS. Cells were then fixed with neutral fixative (Servicebio, Wuhan, China) at room temperature for 30 min. After fixation, cells were washed twice with HBSS and counterstained with DAPI (Beyotime, China) for 5 min at room temperature. Following two additional HBSS washes, samples were stored protected from light. Images were acquired using Leica Stellaris 5 WLL (Leica, Germany) and processed with LAS X software. Quantitative analysis was performed using ImageJ software (*n* = 3 cells, ≥ 30 lysosomes/group; mean ± SD).

### 2.23 Measurement of Lysosomal pH

Lysosomal pH was measured using Lysosensor Yellow/Blue DND‐160 (Invitrogen, USA). MC38 cells were seeded into black 96‐well plates at a density of 1 × 10^4^ cells per well. On the day of measurement, LysoSensor staining solution was prepared in Hank's Balanced Salt Solution (HBSS) at a final concentration of 2 μM and prewarmed to 37°C in the dark. After removal of the culture medium and washing with PBS, cells were incubated with 200 μL of LysoSensor staining solution for 25 min. The staining solution was then removed, cells were washed twice with PBS, and maintained in 100 μL of fresh HBSS. Fluorescence was immediately measured using a SpectraMax i3x microplate reader with excitation at 329/395 nm and emission at 440/540 nm. Fluorescence intensity at 329/440 nm (F1) and 395/540 nm (F2) was recorded, and lysosomal pH was calculated based on the ratio of F2/F1 using a standard calibration curve.

For pH calibration, a standard curve was generated according to previously described protocols [27]. Briefly, after LysoSensor staining, cells were incubated with calibration buffers at pH 3.5, 4.5, 5.5, 6.5, or 7.5 (Bestbio, China) containing 20 μM nigericin (MedChemExpress, USA) for 10 min. Fluorescence measurements were then performed as described above.

### 2.24 Statistical Analysis

Data processing and statistical analysis were performed using GraphPad Prism and R software. Continuous data are presented as mean ± standard deviation (Mean ± SD). The experimental data were not excluded. Normality and homogeneity of variances were assessed using SPSS software. Based on whether the data met the assumptions for parametric tests, appropriate statistical methods were applied: for comparisons between two groups that satisfied normality and homogeneity of variances, unpaired or paired two‐tailed Student's *t*‐tests were used; for comparisons among more than two groups, one‐way or two‐way analysis of variance (ANOVA) was employed. *p*‐values less than 0.05 were considered statistically significant; the level of significance was set at *p* < 0.05 (*), *p* < 0.01 (**), *p* < 0.01 (***) or *p* < 0.001 (****).

## 3 Results

### 3.1 Periodontitis Accelerates Colorectal Cancer Onset and Progression in Mice

To investigate the impact of PD on CRC, we induced periodontitis in *Apc* ^*+/−*^mice by ligating the maxillary second molars. Following ligation, the mice developed significant periodontitis (Figure 1A). H&E staining and Micro‐CT analysis confirmed pronounced alveolar bone loss between the second and third molars in the PD group compared to the sham group (Figure 1B,C). TRAP staining revealed increased osteoclast activity in PD mice (Figure 1D). The distance from the alveolar bone crest (ABC) to the cementoenamel junction (CEJ) between the second and third molars was measured via HE staining and micro‐CT, showing a statistically significant increase in the PD group compared to the sham group (Figure 1E). TRAP staining also revealed a higher number of osteoclasts in the PD group (Figure 1E). These findings indicate that the periodontitis model was established.

**FIGURE 1:**
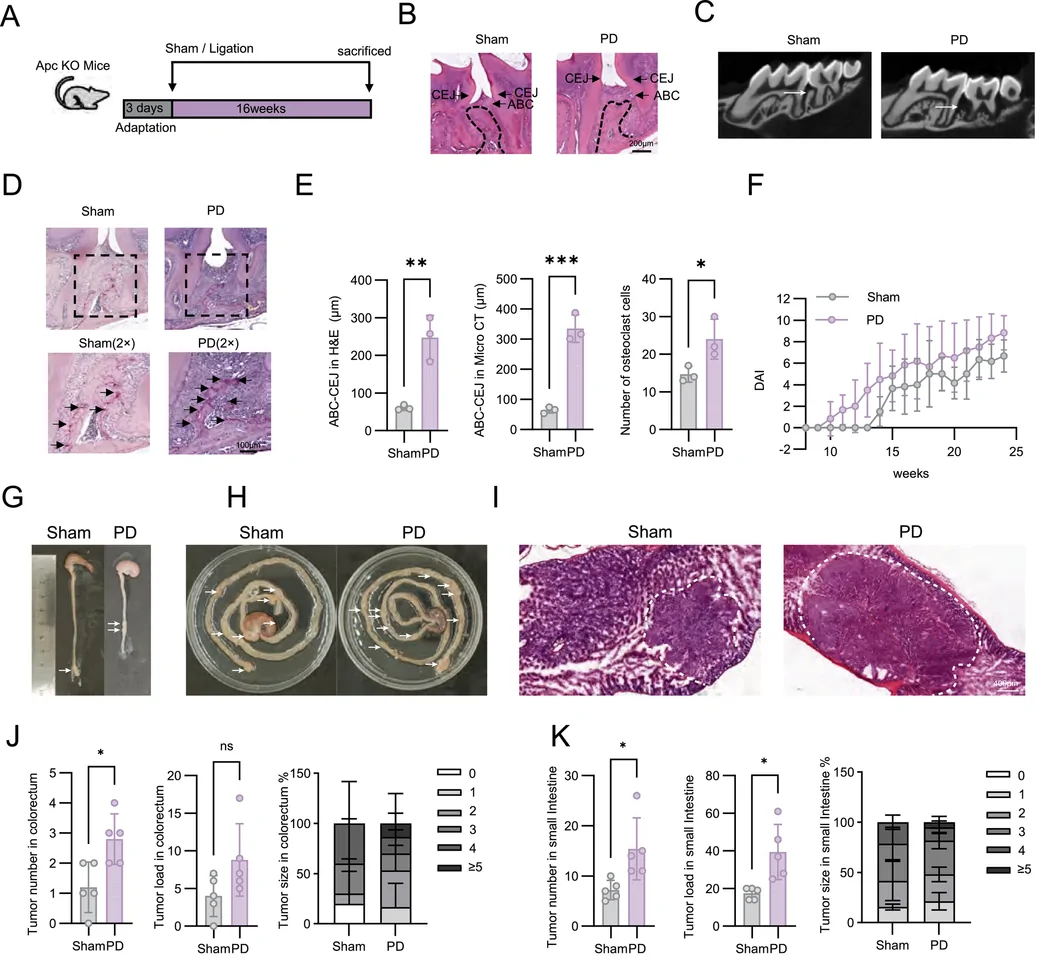
Periodontitis promotes the malignant progression of colorectal cancer in mice. (A) Schematic of animal modelling: Periodontitis was induced in 8‐week‐old *Apc* ^*+/−*^ mice via ligature. Mice were sacrificed at 24 weeks of age. (B) H&E staining of alveolar bone between the second and third maxillary molars in mice. The arrows indicate CEJ and ABC, scale bar = 200 μm. (C) Micro‐CT cross‐sectional view of murine alveolar bone. (D) TRAP staining of alveolar bone between the second and third maxillary molars in mice. The arrows indicate osteoclasts, scale bar = 100 μm. (E) The distance from the alveolar bone crest to the cementoenamel junction in H&E staining and micro‐CT, *n* = 3. Number of osteoclasts of alveolar bone between the second and third maxillary molars in mice, *n* = 3. (F) DAI scoring during tumour progression (8–24 weeks, *n* = 6). (G) Representative colon images of mice (PD or Sham). The arrows indicate the tumour locations. (H) Representative intestine images of mice (PD or Sham). The arrows indicate the tumour locations. (I) Representative H&E staining of tumour in *Apc* ^*+/−*^ mice, scale bar = 400 μm. (J) Tumour number, tumour load and tumour size in the colorectum (*n* = 5). (K) Tumour number, tumour load and tumour size in the small intestine (*n* = 5). Statistical significance：*P＜0.05, **P＜0.01, ***P＜0.001.

Next, symptoms of colorectal tumour development were monitored weekly in mice from the PD and sham groups. PD group exhibited earlier onset of colorectal cancer symptoms, as measured by the disease activity index (DAI), beginning at week 10 compared to week 14 in the sham group (Figure 1F). From weeks 8 to 24, the average DAI scores in the PD group were consistently higher than or equal to those in the sham controls (Figure 1F). At the experimental endpoint (24 weeks of age), tumour burden was assessed in both the colorectum and small intestine. Representative images of tumours and H&E staining are shown in Figure 1G–I. In the colorectum, the PD group developed a significantly greater number of tumours (2.8 ± 0.84 vs. 1.2 ± 0.84; *p* < 0.05) and showed a trend towards increased total tumour load (8.8 ± 4.82 vs. 4.0 ± 2.74; *p* = 0.089) compared to the sham group (Figure 1J). Notably, 13.33% of colorectal tumours in the PD group were ≥ 5 mm in diameter, whereas no tumours of this size were observed in the sham group (Figure 1J). In the small intestine, the PD group also demonstrated significantly increased tumour multiplicity (15.4 ± 6.19 vs. 7.2 ± 1.92; *p* < 0.05) and total tumour load (39.4 ± 14.6 vs. 17.4 ± 3.13; *p* < 0.05) (Figure 1K). Similarly, 4.96% of small intestinal tumours in the PD group reached a size ≥ 5 mm, a feature absent in controls (Figure 1K).

The above results indicate that PD promotes the progression of colorectal cancer in *Apc* ^+/−^ mice.

### 3.2 Inhibition of PDEVs Release Attenuates PD‐Accelerated Tumorigenesis

To investigate the role of PDEVs in CRC development, researchers administered GW4869 intraperitoneally to inhibit EV release as previously described [28] and assessed CRC progression in the PD/PD+GW4869 groups to determine the contribution of PDEVs to intestinal tumorigenesis (Figure 2A). EVs were isolated from PD mice and PD+GW4869 mice plasma; transmission electron microscopy showed typical vesicular morphology (Figure 2B). Nanoparticle tracking analysis (NTA) demonstrated that plasma EVs concentration was lower in GW4869‐treated PD mice than in PD mice (Figure 2C), indicates that GW4869 effectively inhibited the release of circulating EVs in mice. The DAI scores of mice in both groups were recorded throughout the experiment. The PD+GW4869 group exhibited a delayed onset of CRC symptoms at week 12, compared to week 10 in the PD group (Figure 2D). Moreover, the average DAI scores of the PD+GW4869 group were consistently lower than or equal to those of the PD group from weeks 8 to 24 (Figure 2D). GW4869 treatment significantly reduced colorectal tumour burden at the experimental endpoint (Figure 2E). The PD+GW4869 group developed significantly fewer colorectal tumours compared to the PD group (2.0 ± 0.71 vs. 3.6 ± 1.14; *p* < 0.05) (Figure 2G). Although the total tumour load showed a decreasing trend (7.0 ± 2.83 vs. 12.8 ± 6.76; *p* = 0.115), it did not reach statistical significance (Figure 2G). Notably, the proportion of large tumours (≥ 5 mm in diameter) was markedly lower in the PD + GW4869 group (6.67% vs. 29.67%) (Figure 2G). A similar phenotype was observed in the small intestine. Tumour number (13.2 ± 2.78 vs. 19.0 ± 4.36; *p* < 0.05) and total tumour load (30.8 ± 6.54 vs. 55.8 ± 5.93; *p* < 0.001) were both significantly reduced in the PD + GW4869 group compared to the PD group (Figure 2H). The incidence of large tumours (≥ 5 mm) was also lower in the treated group (6.61% vs. 15.49%) (Figure 2I). Representative H&E staining of tumours are shown in Figure 2J.

**FIGURE 2:**
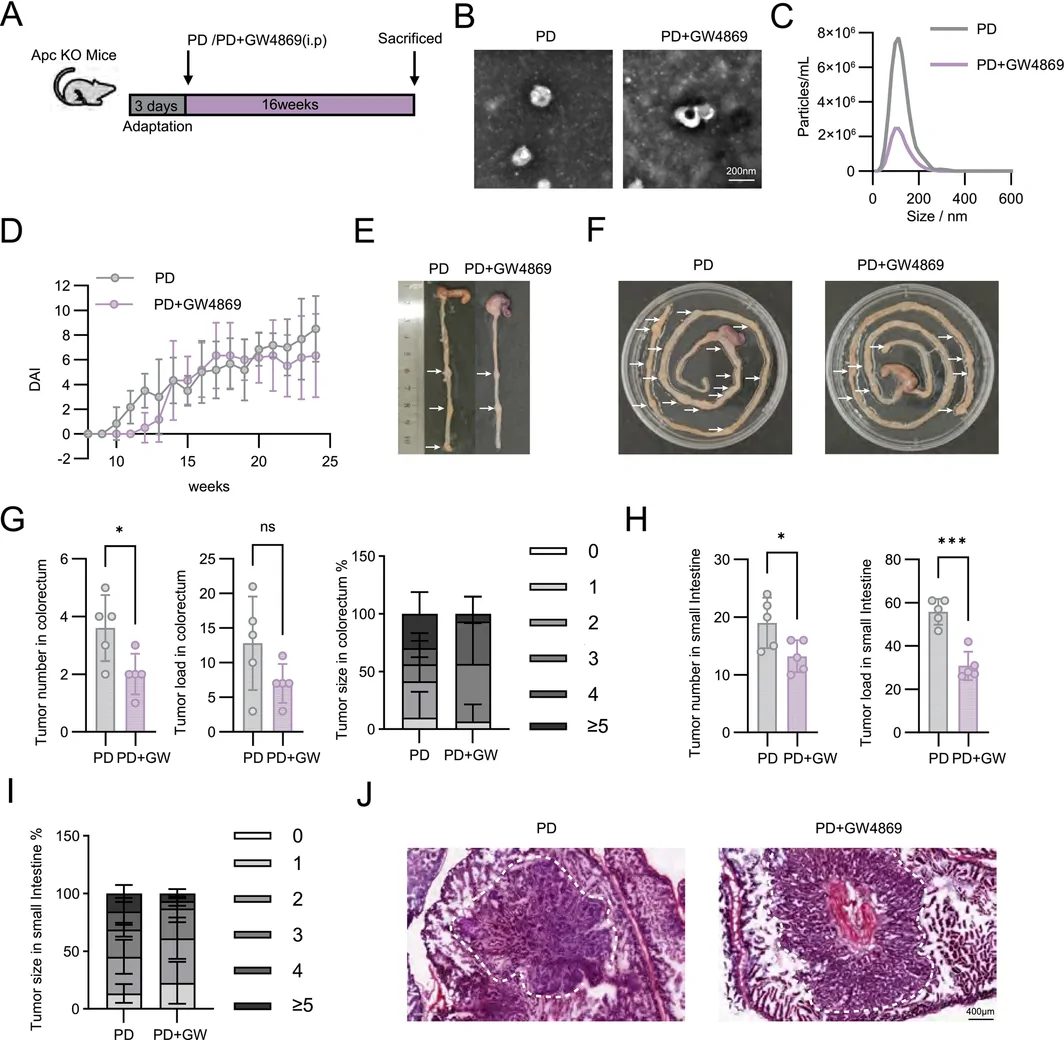
Inhibiting extracellular vesicles (EVs) secretion in periodontitis mice delays the progression of colorectal cancer. (A) Schematic of animal modelling: Periodontitis was induced in 8‐week‐old *Apc* ^*+/−*^ mice. GW4869 was administered via intraperitoneal injection every 2 days. Mice were sacrificed 16 weeks later. (B) Representative electron microscopy images of mice circulation EVs (scale bar = 200 nm). (C) NTA analysis of mice plasma EVs. (D) DAI scoring during tumour progression (8–24 weeks, *n* = 6). (E) Representative colon images of PD or PD + GW4869 mice. The arrows indicate the tumour locations. (F) Representative intestine images of PD or PD + GW4869 mice. The arrows indicate the tumour locations. (G) Tumour number, tumour load and tumour size in the colorectum (*n* = 5). (H) Tumour number and tumour load in the small intestine (*n* = 5). (I) Tumour size in the colorectum (*n* = 5). (J) Representative H&E staining of tumour in *Apc* ^*+/−*^ mice, scale bar = 400 μm. Statistical significance：*P＜0.05, ***P＜0.001.

These results indicate that inhibiting the release of circulating EVs delays CRC progression in PD mice, suggesting that PD‐EVs play a critical role in CRC progression.

### 3.3 PDEVs Promote MC38 Tumour Malignancy In Vivo and In Vitro

To investigate the specific effects of PDEVs on CRC, researchers established a subcutaneous xenograft model using MC38 cells in C57BL/6J mice. According to a dose–response study using three different dosages of PDEVs (10, 20 and 40 μg). The 20 μg dose was selected for the main experiments because it induced a significant pro‐tumorigenic effect without reaching a saturated or excessive level (Figure S1A–C). Peritumoral injections were administered with Vehicle, HEVs/PDEVs (20 μg) (Figure 3A). After successful PD modelling, circulating EVs were isolated from the plasma of sham or PD mice via ultracentrifugation and characterised using WB, NTA, and TEM (Figure 3B–D). The recorded tumour growth curves and final weights revealed that the PDEVs group exhibited significantly faster tumour growth, with both tumour volume (1277 ± 224 mm^3^ vs. 649.5 ± 184.6 mm^3^, *p* < 0.01) and weight (979 ± 325.1 mg vs. 610.4 ± 172.5 mg, *p* < 0.05) showing statistically significant differences compared to the vehicle group at the endpoint of the experiment (Figure 3E,H). Gross morphological observations of the xenografts further supported these findings (Figure 3F). These results collectively indicate accelerated tumour proliferation in the PDEVs‐treated group.

**FIGURE 3:**
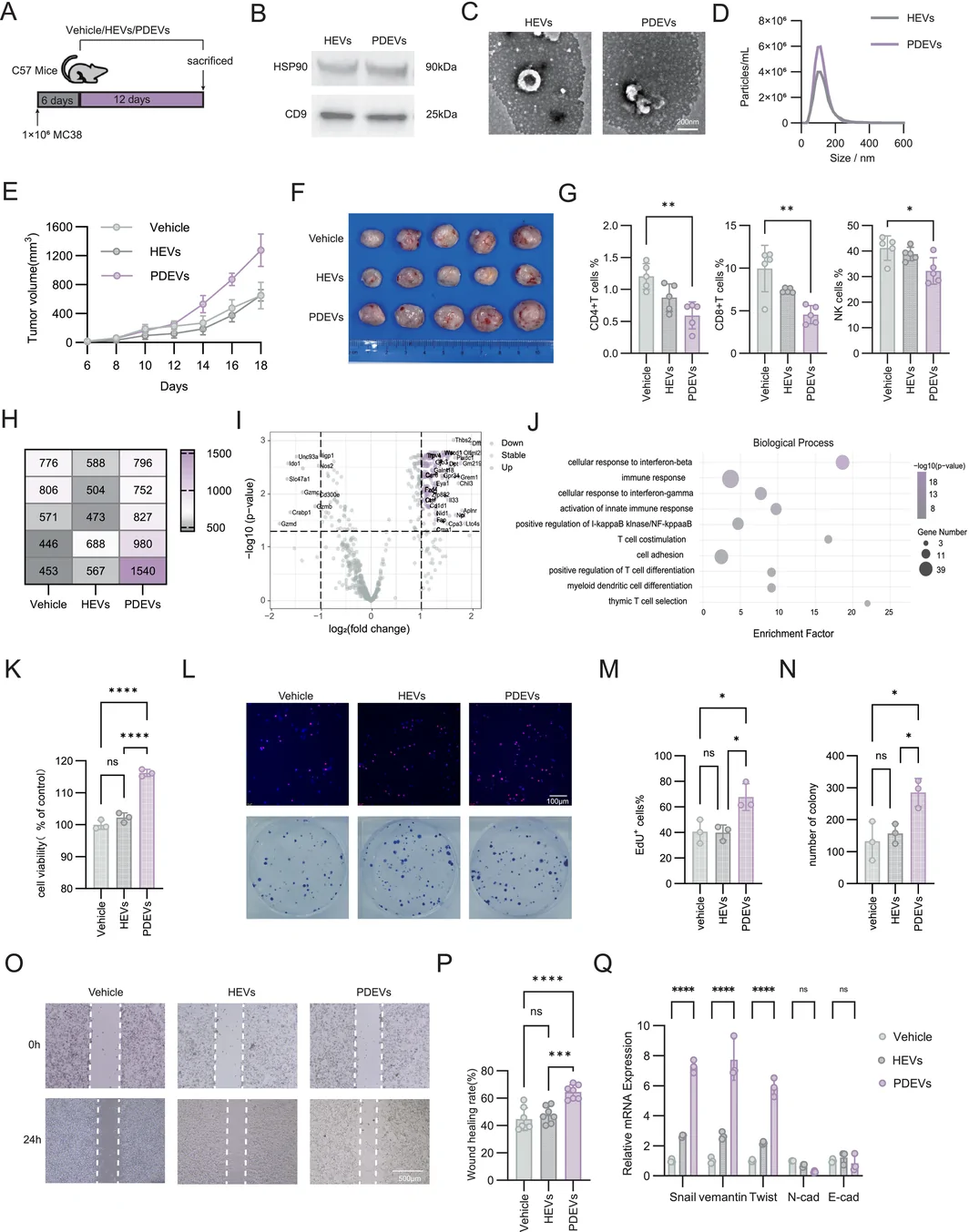
PDEVs promote MC38 malignant phenotypes in vivo and in vitro. (A) Schematic of MC38 subcutaneous tumour model: 1 × 10^6^ MC38 cells were injected into the right flank of male C57BL/6J mice (*n* = 5), from day 6 post‐inoculation vehicle (PBS), HEVs or PDEVs (20 μg per injection, derived from Sham or PD mice) were administered peritumorally every 2 days until the end of the experiment. (B) WB identification of mice circulating EV markers. (C) Representative electron microscopy images of mice circulation EVs (scale bar = 200 nm). (D) NTA analysis of mice circulating EVs. (E) Growth curves of MC38 transplanted tumour volumes (*n* = 5), Data presented as mean ± SD. (F) Representative image of MC38 transplanted tumours in mice (*n* = 5). (G) Proportions of CD4^+^ T cells, CD8^+^ T cells, and NK cells in transplanted tumours by Flow cytometry analysis (*n* = 5). (H) Tumour weight of MC38 transplanted tumours in mice (*n* = 5). (I) Volcano plot of RNA‐seq results (Vehicle vs. PDEVs). (J) RNA‐seq results of transplanted tumours: Gene Ontology (GO) term analysis of biological process. (K) Cell viability % of control by cck8 kit (*n* = 3). (L) Representative images showing EdU staining and colony formation in MC38 cells. (M) Quantification of EdU^+^ cells (%) (mean ± SD, *n* = 3). (N) Quantification of colony numbers (mean ± SD, *n* = 3). (O) MC38 cell migration in wound healing assay (0/24 h). Lines: Initial wound edges (scale bar = 500 μm). (P) Quantification of wound healing rate (mean ± SD, *n* = 7). (Q) RNA expression changes of EMT markers in MC38 cells. (mean ± SD, *n* = 3). Statistical significance：*P＜0.05, **P＜0.01, ***P＜0.001, ****P＜0.0001.

To investigate the impact of PDEVs on immunity within the tumour microenvironment, CD4^+^ T cells, CD8^+^ T cells, and NK cells were selected as research subjects and analysed using flow cytometry. Results revealed that the proportions of CD4^+^ T cells, CD8^+^ T cells, and NK cells among CD45^+^ cells in the xenograft tumours of the PDEVs group were significantly lower than those in the vehicle group (Figure 3G). RNA sequencing of tumour samples was performed, and compared to the vehicle group, the PDEVs group exhibited a distinct profile of differentially expressed genes, with 289 significantly upregulated and 515 downregulated genes (Figure 3I). GO analyses indicated that, compared to the vehicle group, the PDEVs group exhibited alterations in immune‐related biological processes (such as cellular response to interferon‐beta and immune response) (Figure 3J). These results collectively suggest that MC38 xenograft tumours treated with PDEVs are in an immunosuppressed state.

Based on these findings, PDEVs were shown to promote the growth of MC38 xenograft tumours in vivo and to drive an immunosuppressive tumour microenvironment; accordingly, the researchers sought to further investigate, in vitro, how PDEVs affect MC38 cells. The CCK‐8 assay showed that cell viability was significantly higher in the PDEVs group than in the Vehicle and HEVs groups (Figure 3K). EdU staining and colony formation assays further indicated higher cell proliferation in the PDEVs group compared with the Vehicle and HEVs groups (Figure 3L–N). In the wound‐healing assay, the PDEVs group exhibited significantly greater migratory efficiency than the Vehicle and HEVs groups over the same period (Figure 3O,P). Cells from each group were collected for qPCR analysis of epithelial–mesenchymal transition (EMT)‐related markers, showing that *Snail*, *Vimentin*, and *Twist* were upregulated in the PDEVs group compared with the Vehicle group (Figure 3Q), suggesting activation of EMT‐associated transcriptional responses.

Collectively, these results indicate that PDEVs‐treated MC38 cells display enhanced proliferative activity, migratory capacity, and EMT potential, suggesting that PDEVs promote malignant phenotypes in MC38 cells.

### 3.4 Periodontitis‐Associated Circulating EVs Deliver Carnosine to the Mouse Intestine

To investigate the mechanism by which PDEVs promote colorectal cancer, metabolomic profiling was performed on the HEVs and PDEVs described above. Principal component analysis indicated high intra‐group consistency and distinct global metabolomic profiles between the two groups (Figure 4A). Metabolomic analysis of PDEVs revealed a significant enrichment of various bioactive substances compared to HEVs. Notably, carnosine showed a markedly elevated enrichment level (log_2_FC = 30.19) (Figure 4B,C). A recent study reported that carnosine, as a buffering metabolite, alleviates acidity in hepatocellular carcinoma and promotes malignant progression [23]. Next, ELISA was used to quantify carnosine concentrations in EV‐depleted plasma (plasma subjected to ultracentrifugation to remove EVs) and plasma‐derived circulating EVs from sham/PD mice and health/PD humans. The results showed no statistically significant difference in plasma carnosine levels between the two groups, whereas carnosine levels were higher in PDEVs than in the sham/health group (Figure 4D).

**FIGURE 4:**
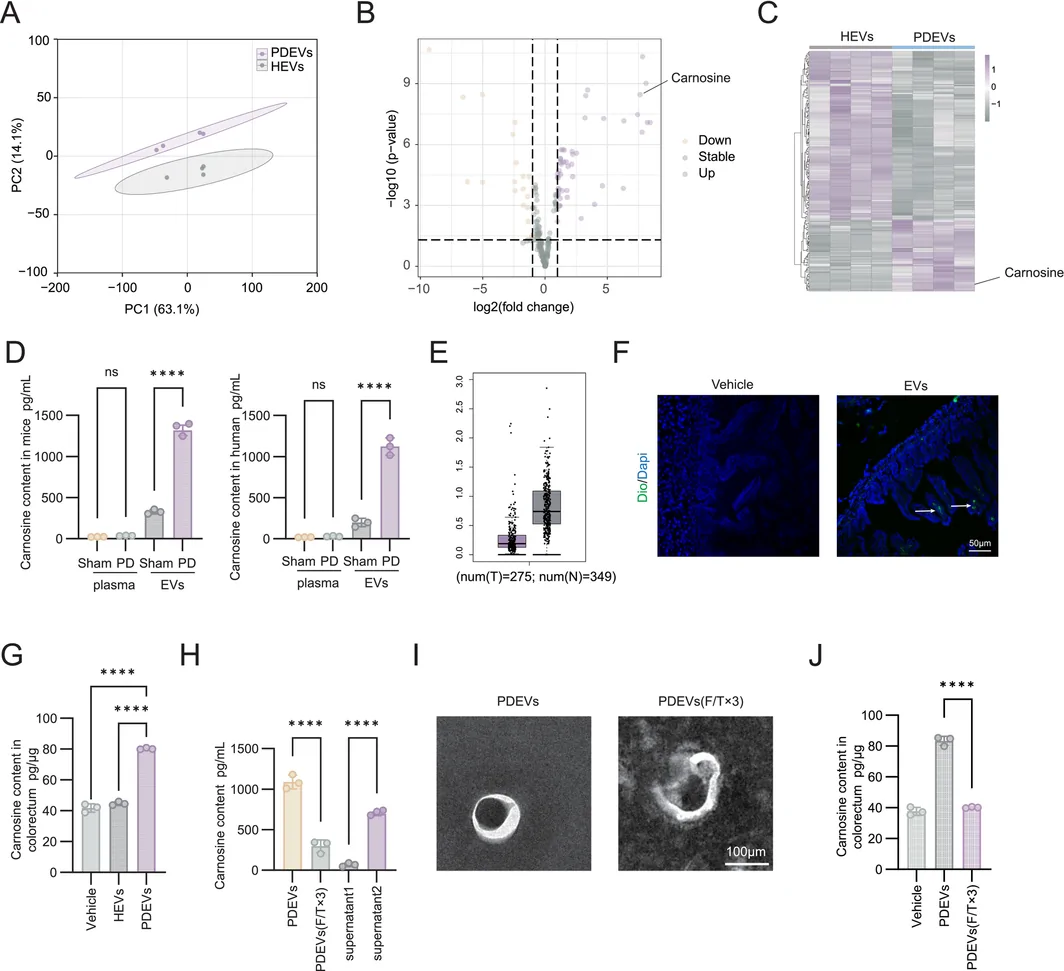
Periodontitis‐associated circulating EVs deliver carnosine to the mouse intestine. (A) PCA plot of metabolite abundance in circulating EVs of mice (PDEVs vs. HEVs). (B) Volcano plot of metabolite abundance in circulating EVs of mice (PDEVs vs. HEVs). (C) Heatmap of metabolite abundance in circulating EVs of mice (PDEVs vs. HEVs). (D) Quantification of carnosine in mice/human plasma and EVs (Sham or PD) by ELISA (*n* = 3), with EV samples normalised to an equal starting volume of the source plasma. (E) Expression of *CARNS1*in TCGA colon adenocarcinoma (COAD) was plotted using GEPIA. Transcripts per million values are shown for tumour (T, purple) and normal (N, grey) samples, (T = 275, *N* = 349). (F) Representative fluorescent imaging of mouse intestinal tissue: EVs: Labelled with Dio, administered via tail vein injection. Timepoint: Tissues collected 2 h post‐administration (scale bar = 50 μm). (G) Quantification of carnosine in mice colorectum by ELISA (*n* = 3). (H) Quantification of carnosine in mice EVs by ELISA (*n* = 3). Supernatant 1 is derived from EVs, and Supernatant 2 is derived from the supernatant of EVs subjected to three freeze–thaw cycles. And the EV samples were normalised to an equal starting volume of the source plasma. (I) Representative electron microscopy images of PDEVs and PDEVs subjected to three freeze–thaw cycles (scale bar = 100 nm). (J) Quantification of carnosine in mice colorectum by ELISA (*n* = 3). Statistical significance：****P＜0.0001.

Carnosine is primarily synthesized in muscle and brain tissues by CARNS1 [23, 29], and based on GEPIA data [30], *CARNS1* is expressed at lower levels in colon cancer tissues than in normal intestinal tissues (Figure 4E), suggesting that carnosine produced intrinsically by intestine tissue is low. To determine whether carnosine is transported to the intestine via PDEVs, researchers conducted the experiment below. First, circulating EVs or PBS (vehicle) were labelled with DiO and injected via tail‐vein in mice. EVs trafficking to the intestinal tract were assessed at 2 h, showing that circulating EVs could reach the intestines (Figure 4F). Next, PBS (vehicle), HEVs, or PDEVs were administered to C57BL/6J mice via tail‐vein injection, and intestinal tissues were collected 2 h later for carnosine quantification. The PDEVs group exhibited higher intestinal carnosine levels than the other two groups (Figure 4G). To further determine whether the carnosine enriched in the intestine was derived from EVs, researchers conducted the experiment below. First, a subset of PDEVs was subjected to three freeze–thaw cycles (F/T × 3) to disrupt vesicle integrity and release their cargo. PDEVs, PDEVs (F/T × 3), and their supernatants were collected for analysis. ELISA showed that carnosine levels in PDEVs (F/T × 3) were lower than those in intact PDEVs (Figure 4H). Transmission electron microscopy revealed that PDEVs subjected to three freeze–thaw cycles displayed disrupted and incomplete structures (Figure 4I). Next, vehicle, PDEVs, or PDEVs (F/T × 3) were administered via tail‐vein injection, and intestinal tissues were harvested 2 h later for ELISA measurement of carnosine. The intestinal carnosine level in the PDEVs (F/T × 3) group was significantly lower than that in the PDEVs group (Figure 4J).

**FIGURE 5:**
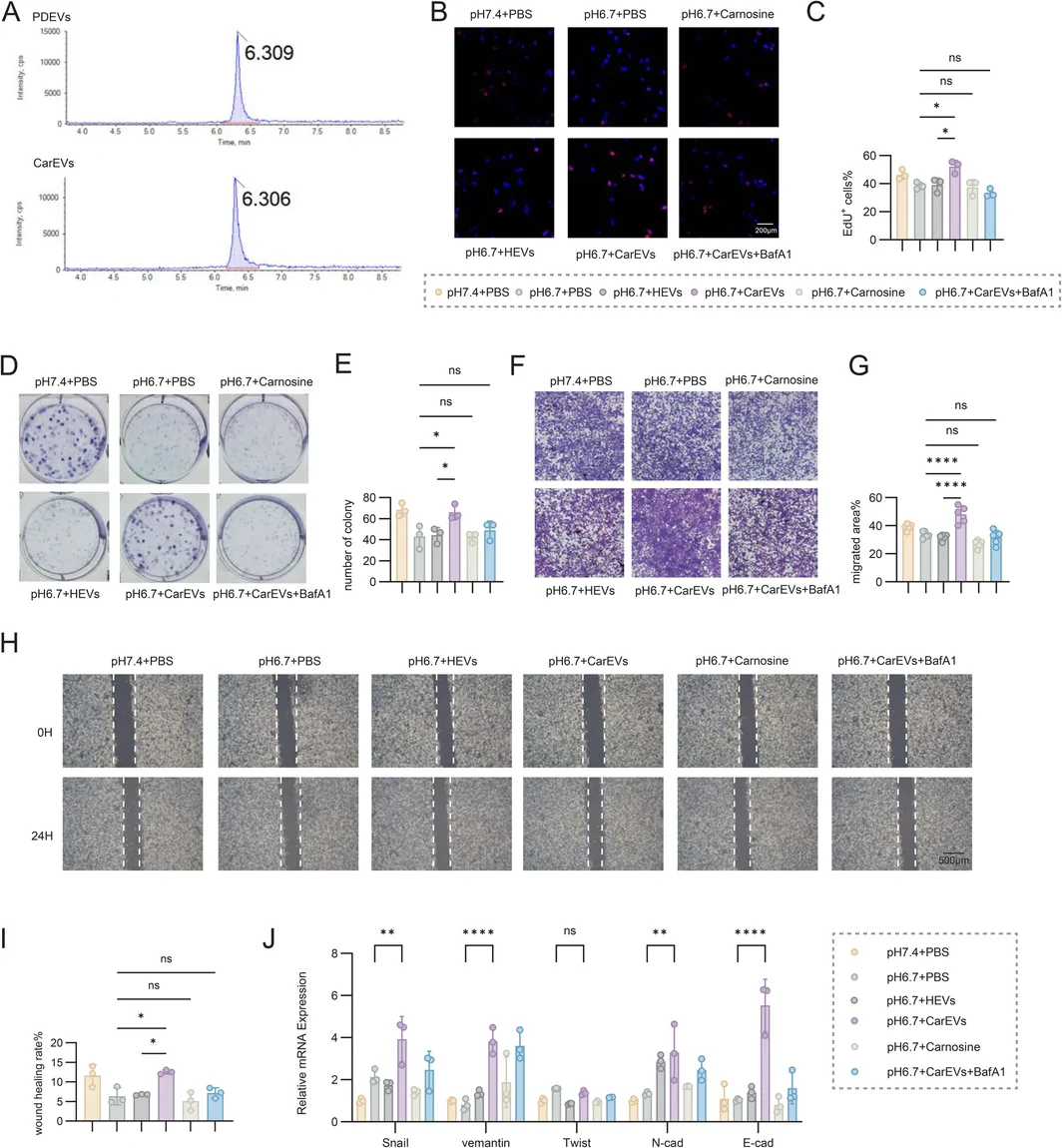
Carnosine^+^ EVs promote malignant progression of MC38 cells. (A) Representative LC–MS chromatograms of carnosine in PDEVs versus CarEVs. (B) Representative images showing EdU staining in MC38 cells. (C) Quantification of EdU^+^ cells (%) (mean ± SD, *n* = 3). (D) Representative images of MC38 cell colony formation. (E) Quantification of colony numbers (mean ± SD, *n* = 3). (F) Representative images of transwell migration of MC38 Cells. (G) Quantification of migrated cells (mean ± SD, *n* = 5). (H) MC38 cell migration in wound healing assay (0/24 h). Lines: Initial wound edges (scale bar = 500 μm). (I) Quantification of wound healing rate (mean ± SD, *n* = 3). (J) RNA expression changes of EMT markers in MC38 cells. (mean ± SD, *n* = 3). Statistical significance：*P＜0.05, ****P＜0.0001.

Collectively, these results indicate that PDEVs are enriched in carnosine and can deliver carnosine to the mouse intestine.

### 3.5 Carnosine‐Enriched EVs Promote Malignant Phenotypes and EMT of MC38 Cells Under Acidic Conditions

Due to the Warburg effect, solid tumours such as colorectal cancer are characterised by an acidic microenvironment [31, 32]. As a buffering metabolite, carnosine promotes malignant progression of hepatocellular carcinoma via the NFX1–Galectin‐9 axis [23]. Therefore, it is hypothesised that carnosine‐enriched EVs may promote malignant progression of colorectal cancer by alleviating the acidic burden in tumour cells. To test this hypothesis, carnosine was loaded into HEVs by incubation, hereafter referred to as ‘CarEVs’, and LC–MS confirmed successful loading of carnosine (Figure 5A). Using MC38 cells as the model, an acidic tumour microenvironment was simulated with DMEM adjusted to pH 6.7 (as described in the Methods). Cells were treated with PBS (vehicle), HEVs, CarEVs, free carnosine and CarEVs+ Bafilomycin A1 to assess subsequent changes in malignant behaviours of MC38 cells. EdU staining and colony formation assays showed that the pH 6.7 + CarEVs group exhibited stronger proliferative capacity than the pH 6.7 + PBS, pH 6.7 + HEVs, pH 6.7 + carnosine and pH 6.7 + CarEVs + Bafilomycin A1 groups (Figure 5B–E). Transwell and wound‐healing assays further demonstrated that migratory capacity in the pH 6.7 + CarEVs group was also higher than that in the four groups described above (Figure 5F–I). Furthermore, to evaluate the impact of CarEVs on EMT, mRNA levels of EMT‐related markers were measured. The results showed that, compared with the four groups described above, the pH 6.7 + CarEVs group exhibited significantly upregulated expression of *Snail*, *Vimentin*, *N‐cadherin* and *E‐cadherin* (Figure 5J), suggesting enhanced migratory, invasive, and metastatic potential. Collectively, these results demonstrate that CarEVs promote the malignant phenotype of MC38 cells under acidic microenvironmental conditions in a lysosome‐dependent manner, while free carnosine did not show this effect.

### 3.6 CarEVs Alleviate Intracellular Acidosis, Restore Lysosomal Function in MC38 Cells and Regulate the NFX1–Galectin‐9 Signalling Axis in MC38

To determine whether CarEVs alleviate the acidic burden in tumour cells, extracellular pH (pHe) and intracellular pH (pHi) were measured. The results showed that the pH 6.7 + CarEVs group exhibited a significantly lower pHe and a significantly higher pHi compared with the pH 6.7 + PBS group (Figure 6A), indicating that CarEVs markedly alleviated intracellular acidosis in MC38 cells. Subsequently, Lysotracker staining was performed to label lysosomes. Lysosomes in the pH 6.7 + CarEVs group were located closer to the nucleus than those in the pH 6.7 + PBS group (Figure 6B,C), suggesting enhanced lysosomal maturation and acidification. Meanwhile, lysosensor staining was used to assess lysosomal pH. The results demonstrated that lysosomes in the pH 6.7 + CarEVs group were more acidic than those in the pH 6.7 + PBS group (Figure 6D), indicating that CarEVs facilitate proper lysosomal acidification. Furthermore, qPCR analysis revealed that higher mRNA expression levels of several lysosome‐associated markers were higher in the pH 6.7 + CarEVs (Figure 6E). Collectively, these results indicate that CarEVs alleviate the acidic burden in MC38 cells and help maintain normal lysosomal function within an acidic tumour microenvironment.

**FIGURE 6:**
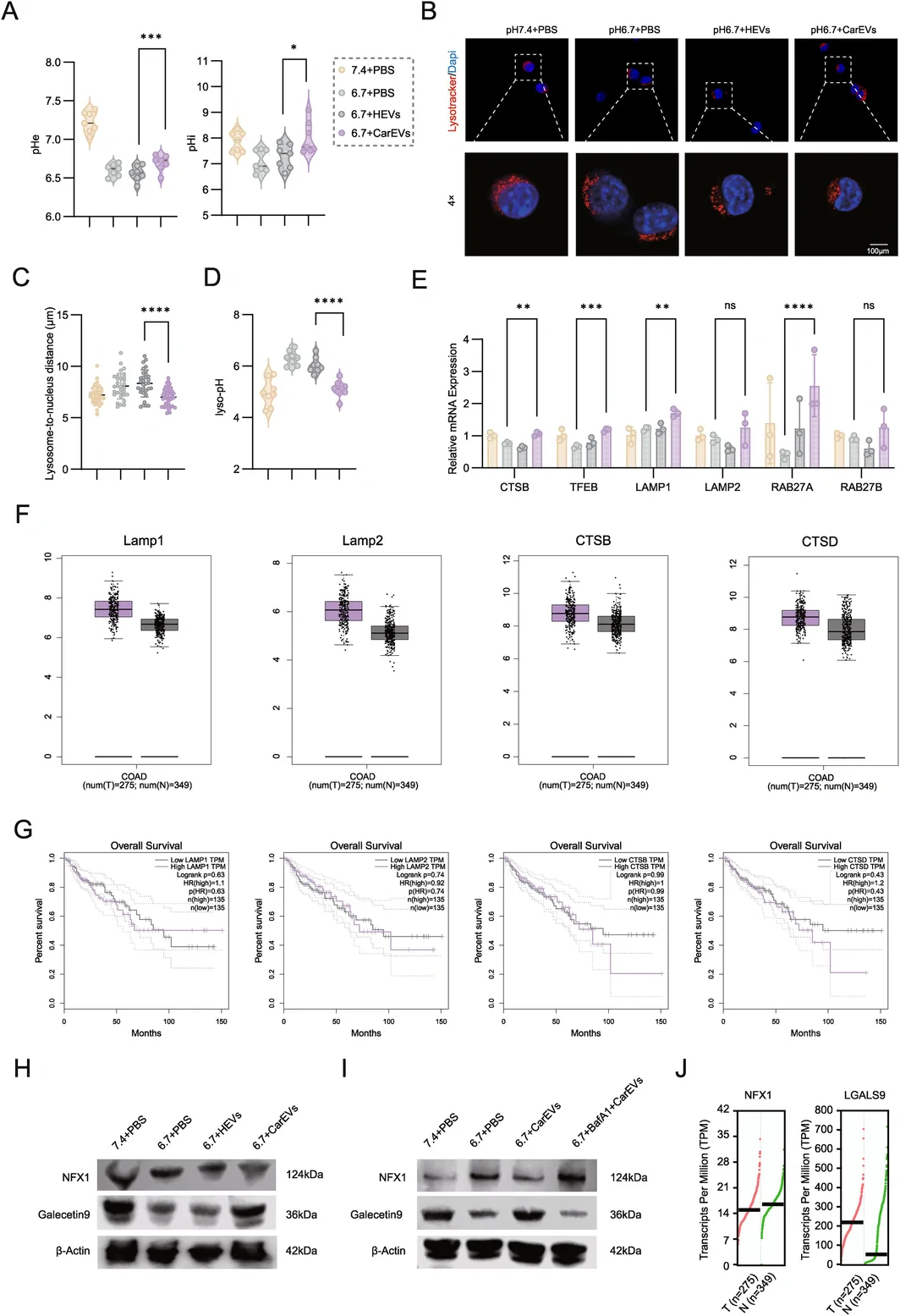
Carnosine^+^ EVs rescue acidosis‐induced lysosomal dysfunction and the clinical significance of lysosomal markers in colorectal cancer. (A) Extracellular pH and Intracellular pH of MC38 cells (mean ± SD, *n* = 7). (B) Representative images of lysosomal staining in MC38 cells (scale bar = 50 μm). (C) Quantitative analysis of lysosome‐to‐nucleus center distances in MC38 cells (*n* = 3 cells, ≥ 30 lysosomes/group; mean ± SD). (D) Quantitative analysis of lysosome pH in MC38 cells (mean ± SD, *n* = 8). (E) RNA expression changes of lysosome markers in MC38 cells. (mean ± SD, *n* = 3). (F) Expression levels of *LAMP1*, *LAMP2*, *CTSB* and *CTSD* in COAD from TCGA database. (G) Correlation between the expression levels of indicated genes (*LAMP1*, *LAMP2*, *CTSB* and *CTSD*) and survival duration in COAD from TCGA database. (H‐I) Protein expression changes of NFX1 and Galectin9 in MC38 cells. (J) Correlation between the expression levels of indicated genes (*NFX1* and *LGALS9*) in COAD from TCGA database. Statistical significance：*P＜0.05, **P＜0.01, ***P＜0.001, ****P＜0.0001.

To further investigate the clinical relevance of lysosomal homeostasis in CRC, researchers analysed the expression and prognostic value of key lysosomal markers (*LAMP1*, *LAMP2*, *CTSB* and *CTSD*) using the TCGA database via GEPIA. Consistent with findings above that CarEVs maintain lysosomal function under acidic stress, it can be observed that these lysosomal genes were significantly upregulated in colon adenocarcinoma (COAD) tissues compared to normal tissues (Figure 6F). Moreover, survival analysis revealed that high expression levels of *LAMP2*, *CTSB* and *CTSD* were significantly correlated with poor prognosis in COAD patients (Figure 6G), supporting the notion that enhanced lysosomal function contributes to COAD progression and poor prognosis.

Previous studies have reported that carnosine promotes the malignant progression of hepatocellular carcinoma through modulation of the NFX1–Galectin‐9 axis [23]. To investigate whether CarEVs regulate the NFX1–Galectin‐9 axis in MC38 cells, cells were treated with PBS or EVs at different pH values. Western blot analysis showed that exposure to pH 6.7 resulted in upregulation of NFX1 and downregulation of Galectin‐9 expression compared with the pH 7.4 control. Notably, treatment with CarEVs under acidic conditions (pH 6.7) markedly decreased the protein levels of NFX1 and increased Galectin‐9 expression compared with both the pH 6.7 + PBS and pH 6.7 + HEVs groups (Figure 6G). To further examine whether the effect of CarEVs on the NFX1–Galectin‐9 axis is dependent on intracellular processing, cells were treated with BafA1. Western blot analysis revealed that BafA1 treatment significantly attenuated the CarEVs‐induced downregulation of NFX1 and upregulation of Galectin‐9 under acidic conditions (Figure 6I), indicating that the regulatory effects of CarEVs are at least partially dependent on lysosome‐related pathways. Last, we analysed the expression and prognostic value of *NFX1* and *LGALS9* in the TCGA database using GEPIA. The results showed that NFX1 expression was lower, whereas LGALS9 expression was higher, in COAD tissues than in normal tissues (Figure 6J).

## 4 Discussion

Periodontitis is a chronic inflammatory disease that has been implicated in a wide range of systemic diseases. However, the mechanisms by which it promotes CRC progression remain poorly understood. It is demonstrated that periodontitis accelerates the initiation and progression of CRC in *Apc* ^*+/−*^ mice via circulating extracellular vesicles. In addition, PDEVs, enriched in the buffering metabolite carnosine, are identified as a key mediator linking oral inflammation to the malignant progression of intestinal tumours.

Using a periodontitis model, it is found that periodontitis leads to an earlier onset of CRC and significantly increases tumour burden of tumours in the colorectum and small intestine. Inhibition of EVs release with GW4869 delays tumour progression and reduces tumour burden, functionally demonstrating the requirement of circulating EVs in periodontitis‐promoted tumour progression. These results indicate that EVs constitute a critical long‐range communication system through which PD influences CRC.

Previous studies on the association between PD and CRC primarily focused on the contribution of oral pathogenic bacteria through their migration to and colonization of the gastrointestinal tract. Bacterial colonization can promote tumour‐associated inflammation. However, EVs also represent a rapid and efficient mode of information transfer. Circulating EVs can broadly disseminate their biological cargo via the bloodstream and lymphatic drainage, thereby exerting prompt effects on distant organs. This finding extends the existing research paradigm and provides an additional EV‐mediated mechanism underlying periodontitis–CRC regulation.

A major challenge in this research area is distinguishing the specific effects of EVs from the broader systemic consequences of periodontitis (e.g., chronic inflammation and immune dysregulation). To address this issue, an MC38 syngeneic transplantation tumour model in immunocompetent C57BL/6J mice is employed. The results indicate that, in the absence of oral infection or pre‐existing systemic inflammation, administration of circulating EVs derived from periodontitis mice alone is sufficient to accelerate tumour growth and induce an immunosuppressive tumour microenvironment. Specifically, reduced infiltration of CD4^+^ T cells, CD8^+^ T cells, and NK cells is observed in tumour tissues, accompanied by alterations in immune‐related transcriptional programs, suggesting that circulating EVs can modulate antitumor immune responses.

In addition to immune regulation, a previously underappreciated metabolic mechanism by which PDEVs promote CRC progression is also revealed. Metabolomic profiling indicates that carnosine is one of the metabolites significantly enriched in PDEVs which function as a metabolic buffer. Solid tumours, including CRC, commonly exhibit an acidic tumour microenvironment driven by aerobic glycolysis [33, 34], which imposes substantial selective pressure on tumour cells.

Notably, the role of lysosomal function in cancer is context‐dependent. In early tumorigenesis, the integrity of the lysosome‐autophagy system often exerts a tumour‐suppressive effect. Conversely, impaired lysosomal degradation may also indirectly promote tumour progression and metastasis by rerouting multivesicular bodies to the plasma membrane, thereby enhancing the release of extracellular vesicles that remodel the tumour microenvironment [35]. However, in established or advanced‐stage tumours, the situation changes. Cancer cells often upregulate lysosomal biogenesis, acidification, and autophagy‐lysosomal flux to adapt to hypoxic and acidic stress, maintain metabolic fitness, and promote invasion. In this setting, hyperactive lysosomal function is frequently co‐opted to support tumour progression [36]. In this study, colorectal cancer represents a highly acidic solid tumour model where cancer cells endure a significant metabolic acid load. When tumour cells internalise PDEVs, the delivered carnosine acts as a potent buffering metabolite. This relieves intracellular acid stress and restores lysosomal homeostasis, which in turn supplies essential nutrients and sustains the malignant phenotypes (e.g., invasion and migration) of MC38 cells.

This study still has several limitations, although it also has some strengths. A major strength of the current work is the use of the *Apc* ^*+/−*^ genetic CRC model, which partially recapitulates the spontaneous development of intestinal tumours and is therefore suitable for investigating the impact of periodontitis on CRC progression in vivo. However, the in vivo evidence is currently restricted to this single model, and further validation in additional models, such as the AOM/DSS‐induced CRC model, is warranted to improve the generalizability of our findings. In addition, although the *Apc* ^*+/−*^ model is valuable for studying genetically driven intestinal tumorigenesis, it cannot fully reflect the heterogeneity and complexity of human CRC.

Another limitation is that the tissue origin of carnosine‐enriched circulating EVs under periodontitis conditions remains unclear. Given that carnosine is primarily synthesized by carnosine synthase 1 in skeletal muscle and brain tissues [37], we speculate that periodontitis‐induced systemic inflammation may trigger host metabolic reprogramming, thereby promoting the redistribution of carnosine in the form of EVs.

Our findings raise the possibility that circulating EV‐associated carnosine may serve as a potential mediator linking periodontitis to CRC progression, and may also have value as a biomarker for systemic metabolic changes in affected patients. However, its clinical relevance in humans remains to be established. Future studies should therefore evaluate circulating EV‐associated carnosine levels in patients with periodontitis and CRC, and determine whether they correlate with disease severity, tumour progression, or clinical outcome.

In summary, a regulatory ‘oral–circulating EVs–intestinal tumor’ axis is identified, elucidating a previously unrecognised mechanism by which periodontitis promotes colorectal cancer progression. Although further clinical validation is required, circulating EV–associated carnosine may serve as a potential biomarker for CRC risk stratification in patients with periodontitis, and targeting EVs release or tumour metabolic adaptation may provide new therapeutic avenues for CRC intervention.

## Author Contributions

**Ruoyi Wu**, **Zihan Cai** and **Hualing Sun:** contributed to conceptualization, data curation, formal analysis and writing original draft. **Haikun Yu**, **Bicheng Zhang** and **Chi Zhang:** contributed to investigation and formal analysis. **Yi Fan**, **Xiaoxuan Zhu**, **Yueqi Ni**, **Yu Cui**, **Kaixin Wang**, **Zhe Li** and **Xinyi Zhou:** contributed to investigation. **Qing He**, **Yanru Wu** and **Yufeng Zhang:** contributed to supervision, project administration. All authors gave final approval and agree to be accountable for all aspects of the work.

## Funding

The authors disclosed receipt of the following financial support for the research, authorship, and/or publication of this article: This work was supported by the National Natural Science Foundation of China (no. 82072483, 82571006 and 82170277), the National Science Fund for Distinguished Young Scholars (No. 82025011).

## Ethics Statement

All animal experiments were conducted in strict compliance with the Guidelines for the Care and Use of Laboratory Animals (Ministry of Science and Technology of China, 2006) and institutional ethical regulations. The experimental protocols were approved by the Institutional Animal Care and Use Committee of the Medical Research Institute of Wuhan University. The ethics approval number is MRI2024‐LAC182. Studies involving human participants were approved by the Ethics Committee of the School and Hospital of Stomatology, Wuhan University, and informed consent was obtained from all participants.

## Conflicts of Interest

The authors declare no conflicts of interest.

## Supporting information


**Figure S1:** PDEVs accelerate MC38 tumour growth in a dose‐dependent manner. (A) Growth curves of MC38 transplanted tumour volumes (*n* = 5), Data presented as mean ± SD. (B) Representative image of MC38 transplanted tumours in mice (*n* = 5). (C) Tumour weight of MC38 transplanted tumours in mice (*n* = 5).

## Data Availability

Source data and reagents are available from the corresponding author upon reasonable request.
