# Supplementary material for: Periodontitis‐Associated Circulating EVs Promote Colorectal Cancer Progression via Carnosine‐Mediated Acidosis Adaptation
Source: Cell Prolif. 2026 Jul 1;59(11):e70254. doi: 10.1111/cpr.70254 (PMC13326038; doi:10.1111/cpr.70254)
Supplement: Supplementary file 1 [file CPR-59-e70254-s001.pdf]

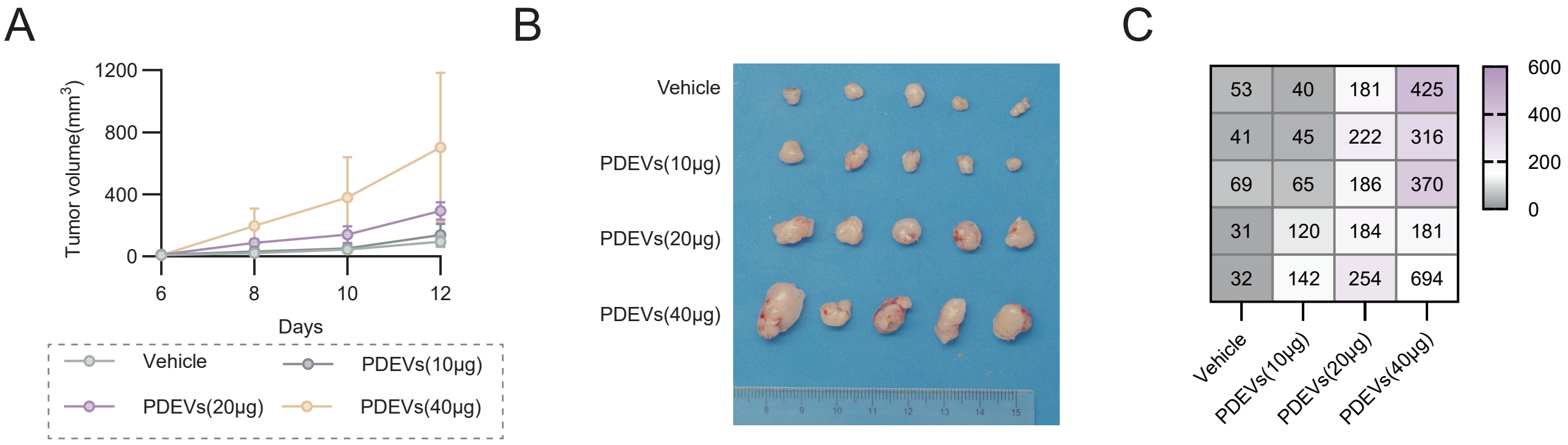
A
1200
800
400
0
Tumor volume(mm³)
6
8
10
12
Days
Vehicle
PDEVs(10μg)
PDEVs(20μg)
PDEVs(40μg)
B
Vehicle
PDEVs(10μg)
PDEVs(20μg)
PDEVs(40μg)
C
53 40 181 425
41 45 222 316
69 65 186 370
31 120 184 181
32 142 254 694
600
400
200
0
Vehicle
PDEVs(10μg)
PDEVs(20μg)
PDEVs(40μg)
