# Supplementary figures and images for: Periodontitis‐Associated Circulating EVs Promote Colorectal Cancer Progression via Carnosine‐Mediated Acidosis Adaptation
Source: Cell Prolif. 2026 Jul 1:e70254. Online ahead of print. doi: 10.1111/cpr.70254 (PMC13326038; doi:10.1111/cpr.70254)

A

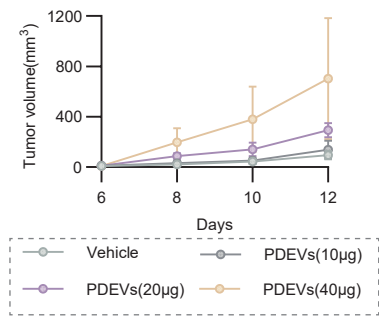

B

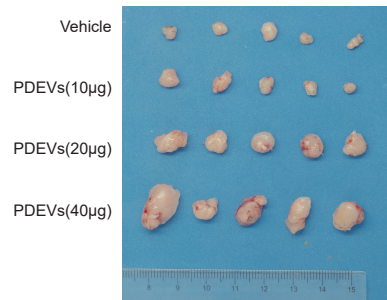

C

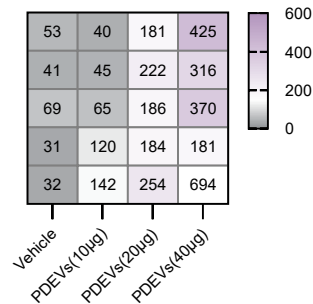

Supplement: Supplementary file 1 — Figure S1: PDEVs accelerate MC38 tumour growth in a dose‐dependent manner. (A) Growth curves of MC38 transplanted tumour volumes (n = 5), Data presented as mean ± SD. (B) Representative image of MC38 transplanted tumours in mice (n = 5). (C) Tumour weight of MC38 transplanted tumours in mice (n = 5). [file CPR-9999-e70254-s001.pdf]
